# Supplementary material for: Triptolide, a Cancer Cell Proliferation Inhibitor, Causes Zebrafish Muscle Defects by Regulating Notch and STAT3 Signaling Pathways
Source: Int J Mol Sci. 2024 Apr 25;25(9):4675. doi: 10.3390/ijms25094675 (PMC11083231; doi:10.3390/ijms25094675)
Supplement: Supplementary file 1 [file ijms-25-04675-s001.zip › ijms-2929531-supplementary.pdf]

**Supplementary Table S1**. Compounds with inhibition of Notch1 signaling identified in the screening.

| | Drug name | Catalog No. |
|---|---|---|
| **Natural compounds** | Magnolol | S2321 |
| | Triptolide | S3604 |
| **FDA approved chemical compounds** | Carmofur | S2189 |
| | Sorbitol | S2393 |
| | Mitoxantrone 2HCl | S2485 |
| | Gemcitabine | S1714 |
| | Terbinafine | S1725 |
| | Mifepristone | S2606 |
| | Prednisolone | S1737 |
| | Teniposide | S1787 |
| | Ouabain | S4016 |
| | Esomeprazde sodium | S2233 |
| | Clomifene citrate | S2561 |
| | Daunorubicin HCl | S3035 |

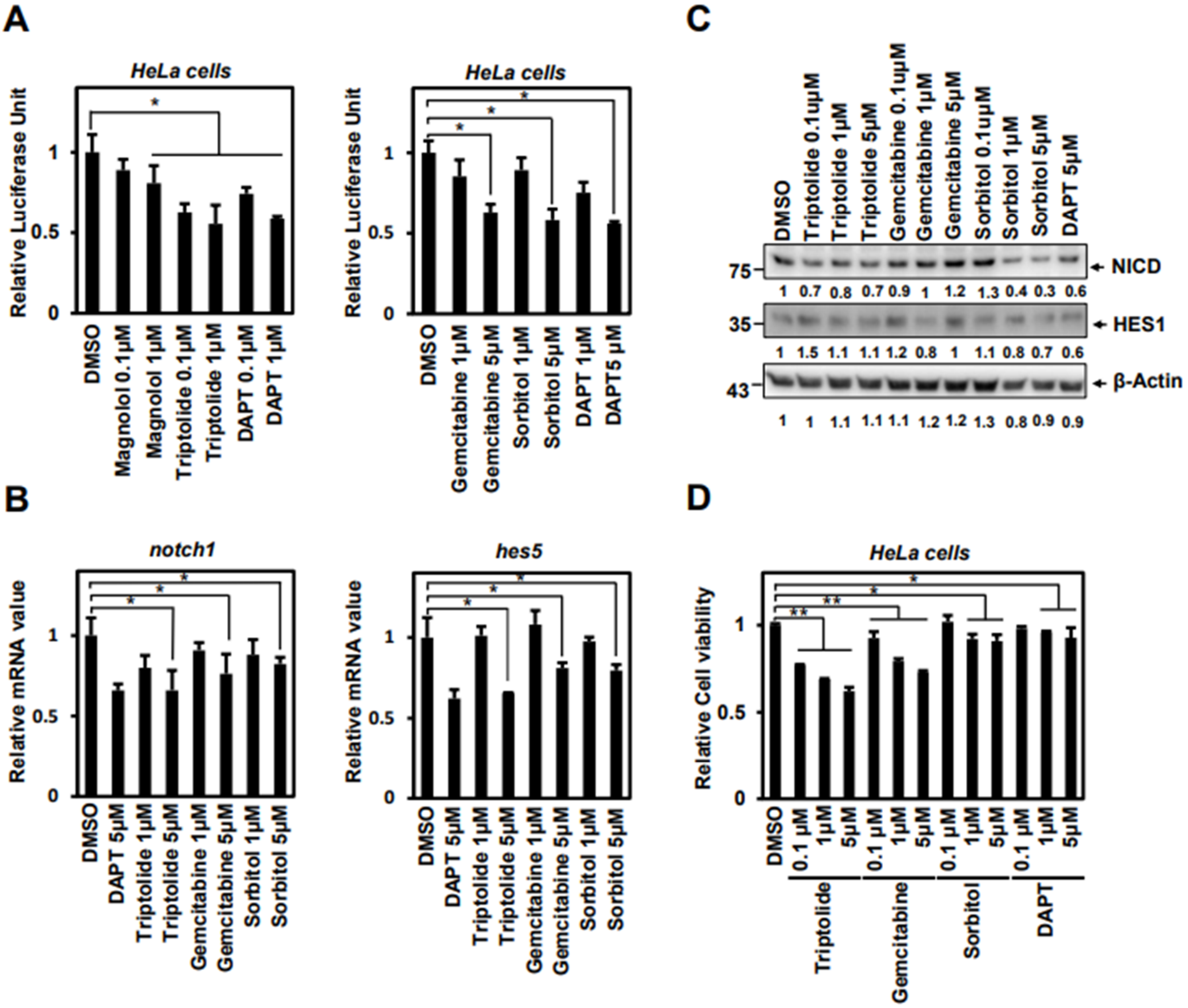


**Supplementary Figure S1.** Selective chemicals inhibit Notch1 signaling in HeLa cells.

HeLa cells were treated with 0.1µM, 1µM, or 5µM concentration of Magnolol, Triptolide, Gemcitabine, Sorbitol, DAPT or DMSO (control)for 24h. (A) Cells were lysed and subjected to a luciferase assay. The luciferase reporter activity in each sample was normalized to Renilla protein activity. (B) Cells were harvested and Total RNA was isolated and subjected to qRT-PCR analysis. Data were normalized to β-Actin expression. (C) Treated cell lysates were subjected to Western blotting with antibodies against NICD, HES1, and β-Actin. We used ImageJ software (NIH, Bethesda, NY, USA) to analyze the membranes. (D) HeLa cells were treated with control (DMSO) or respective concentrations of 100 nM, 1◎M, and 5◎◎M for Gemcitabine, Sorbitol, DAPT and TP. Cells were cultured for 48 hours. Cell viability was measured by MTT assay in 24-well plates. The results represent the means ± S.D. of three independent experiments performed in triplicate. *, P<0.05; **, P<0.01.
